# Supplementary material for: Design, Synthesis and Pharmacological Evaluation of Novel C2,C3-Quinoxaline Derivatives as Promising Anxiolytic Agents
Source: Int J Mol Sci. 2022 Nov 19;23(22):14401. doi: 10.3390/ijms232214401 (PMC9696749; doi:10.3390/ijms232214401)

International Journal of
Molecular Sciences

MDPI

NMR spectra of amines 2b,d,f,g, 4b.

2-(2-((3-(4-(*tert*-Butyl)phenyl)quinoxalin-2-yl)methyl)-4,5-dimethoxyphenyl)-N-methylethanamine hydrochloride (**2b**):

^{1}H NMR spectrum

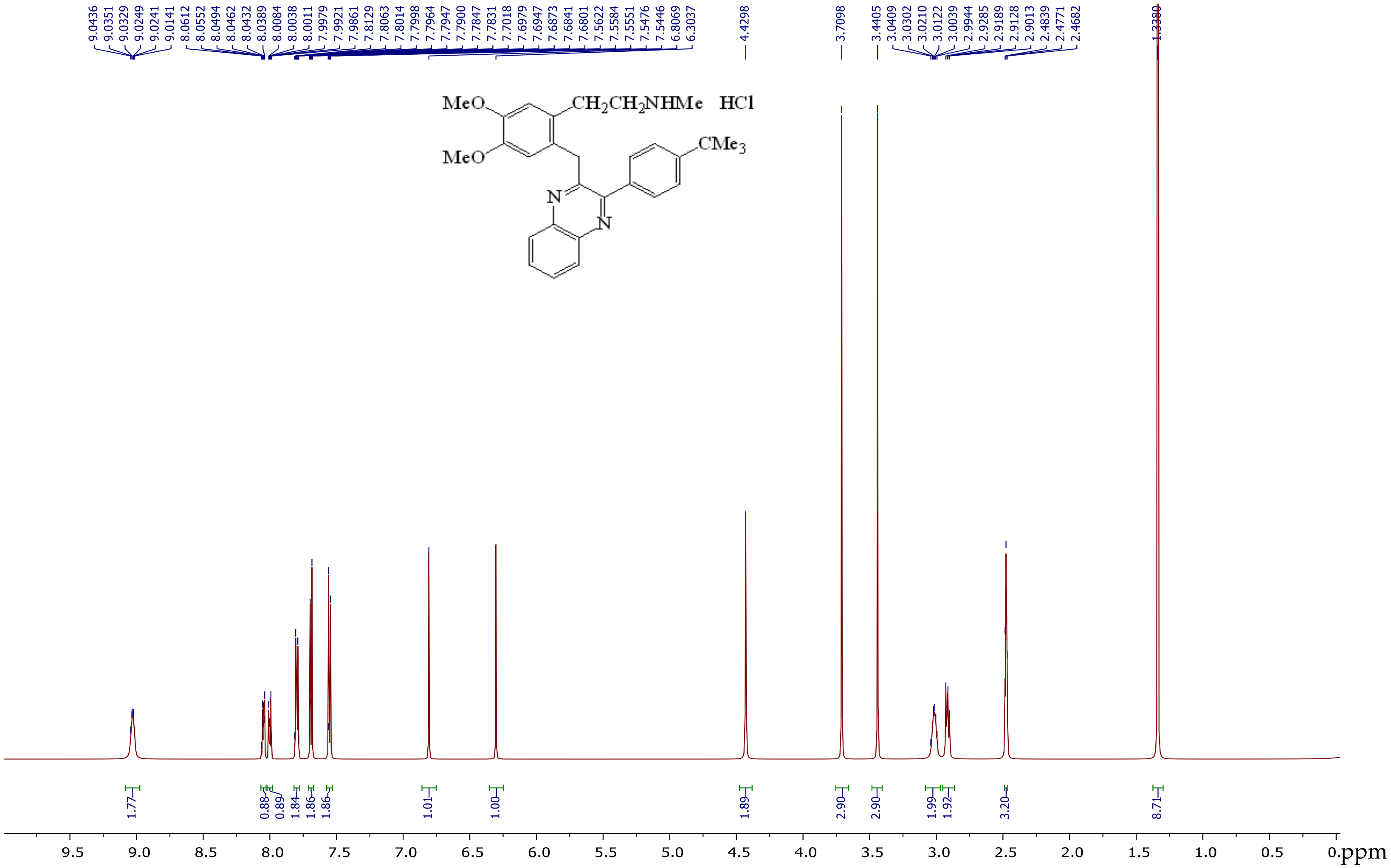

^{13}C NMR spectrum

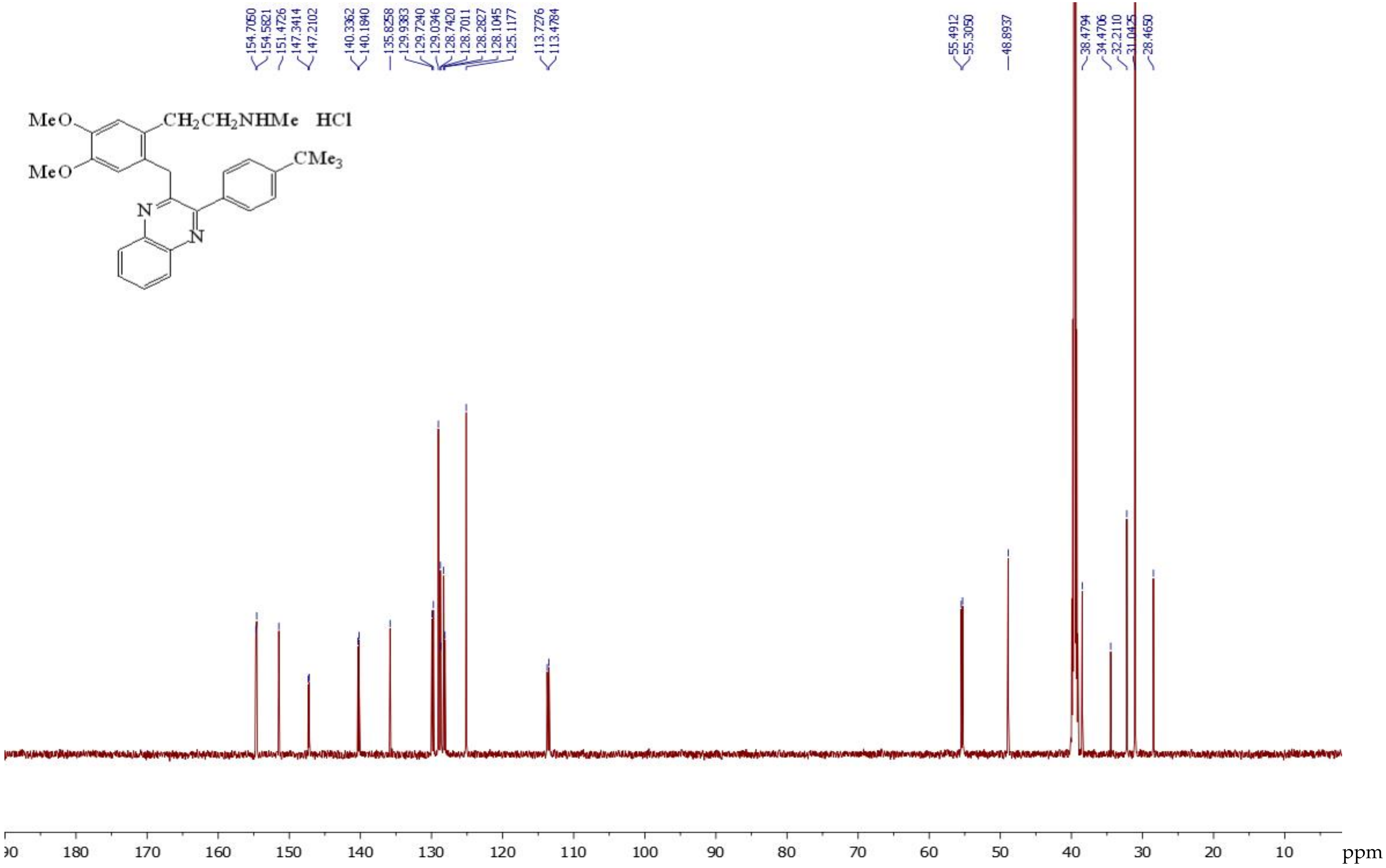

2-(2-((3-(3,4-Dichlorophenyl)quinoxalin-2-yl)methyl)-4,5-dimethoxyphenyl)-N-methylethanamine hydrobromide (**2d**):

^{1}H NMR spectrum

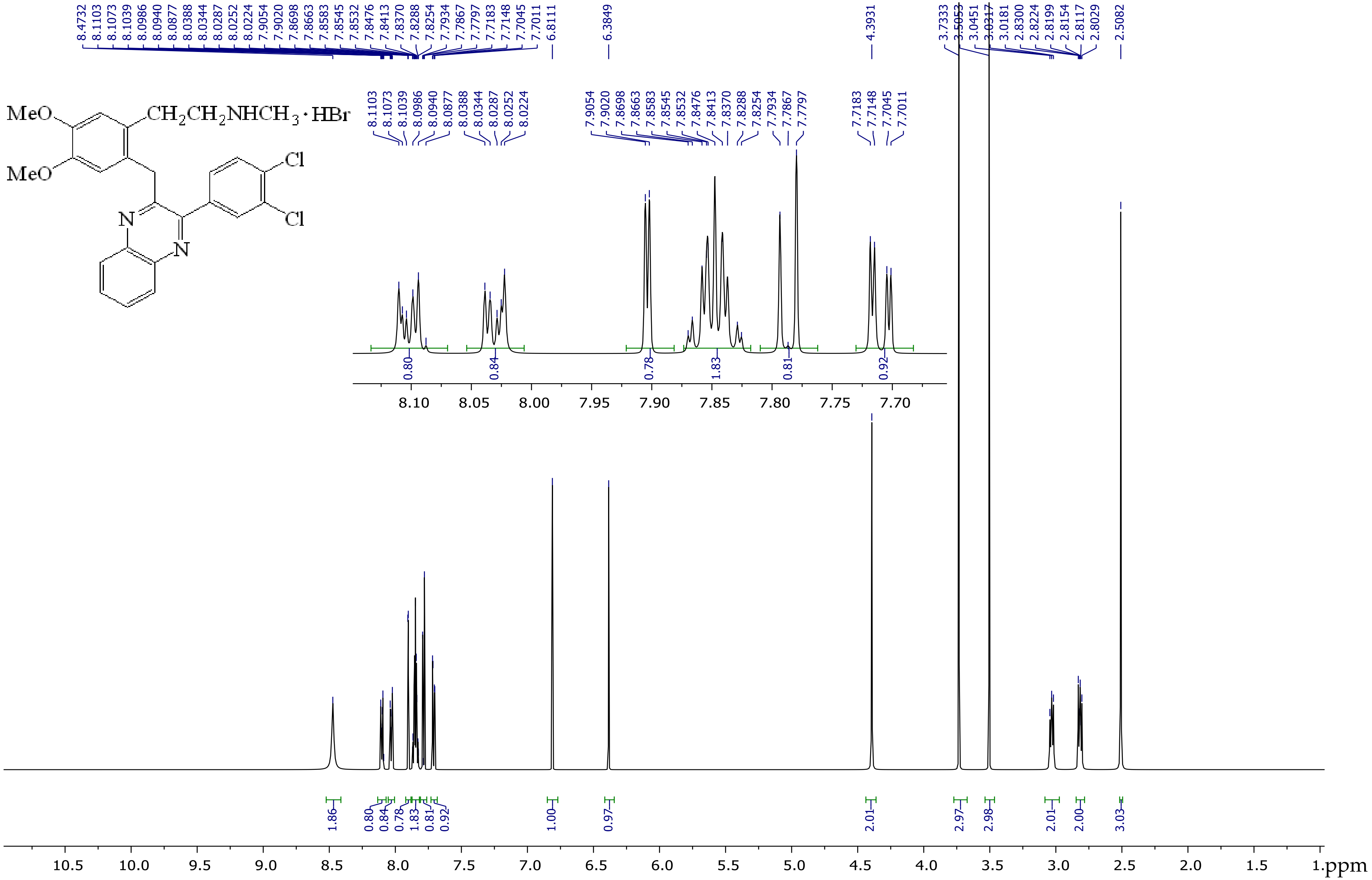

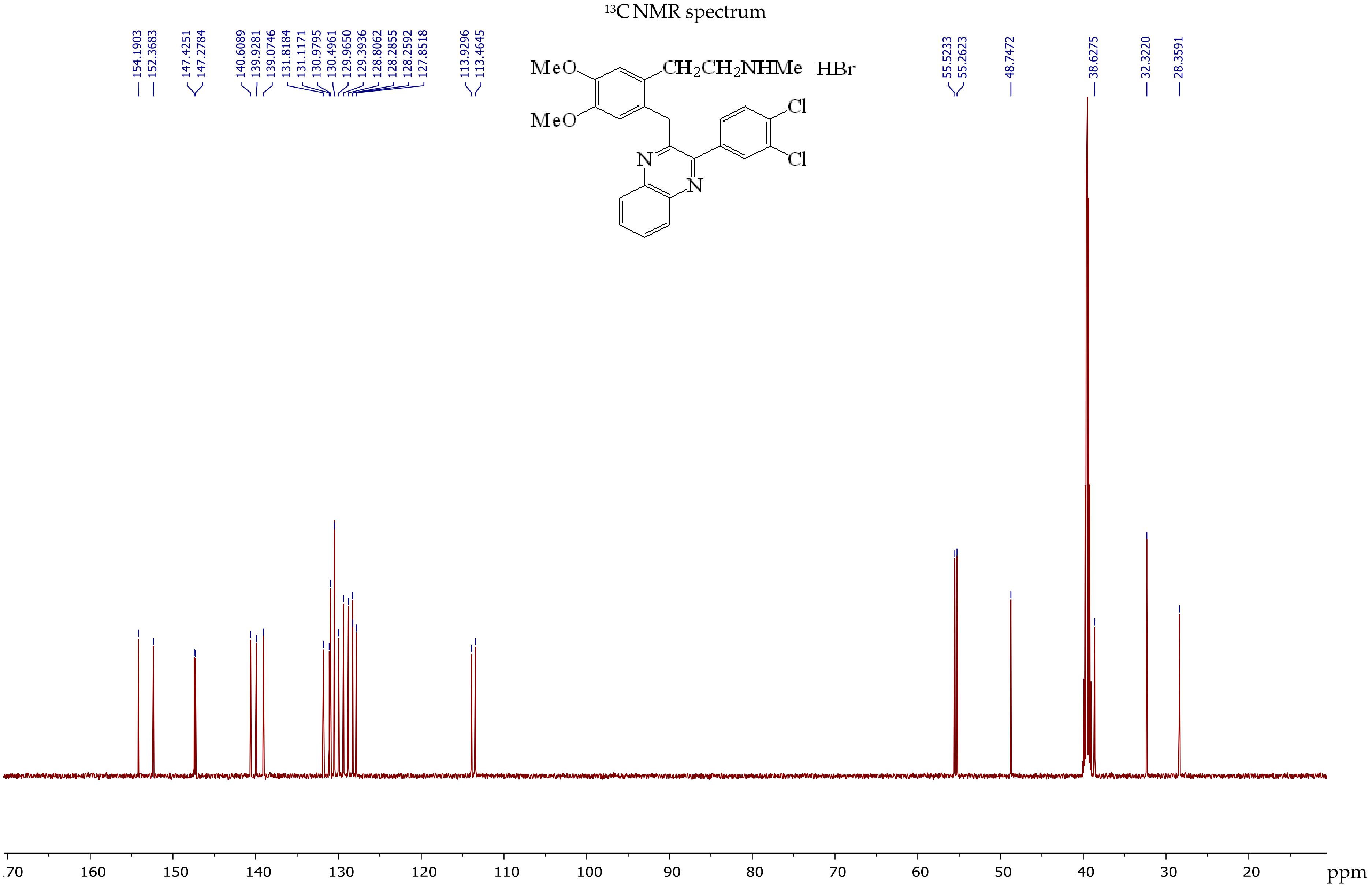

^{13}C NMR spectrum
154.1903
152.3683
147.4251
147.2784
140.6089
139.9281
139.0746
131.8184
131.1171
130.9795
130.4961
129.9650
129.3936
128.8062
128.2855
128.2592
127.8518
113.9296
113.4645
55.5233
55.2623
48.7472
38.6275
32.3220
28.3591
MeO
MeO
CH2CH2NHMe HBr
Cl
Cl
N
N
170
160
150
140
130
120
110
100
90
80
70
60
50
40
30
20
ppm

1-(4,5-Dimethoxy-2-((3-methylquinoxalin-2-yl)methyl)phenyl)-*N*,2-dimethylpropan-2-amine hydrochloride (**2f**):

^{1}H NMR spectrum

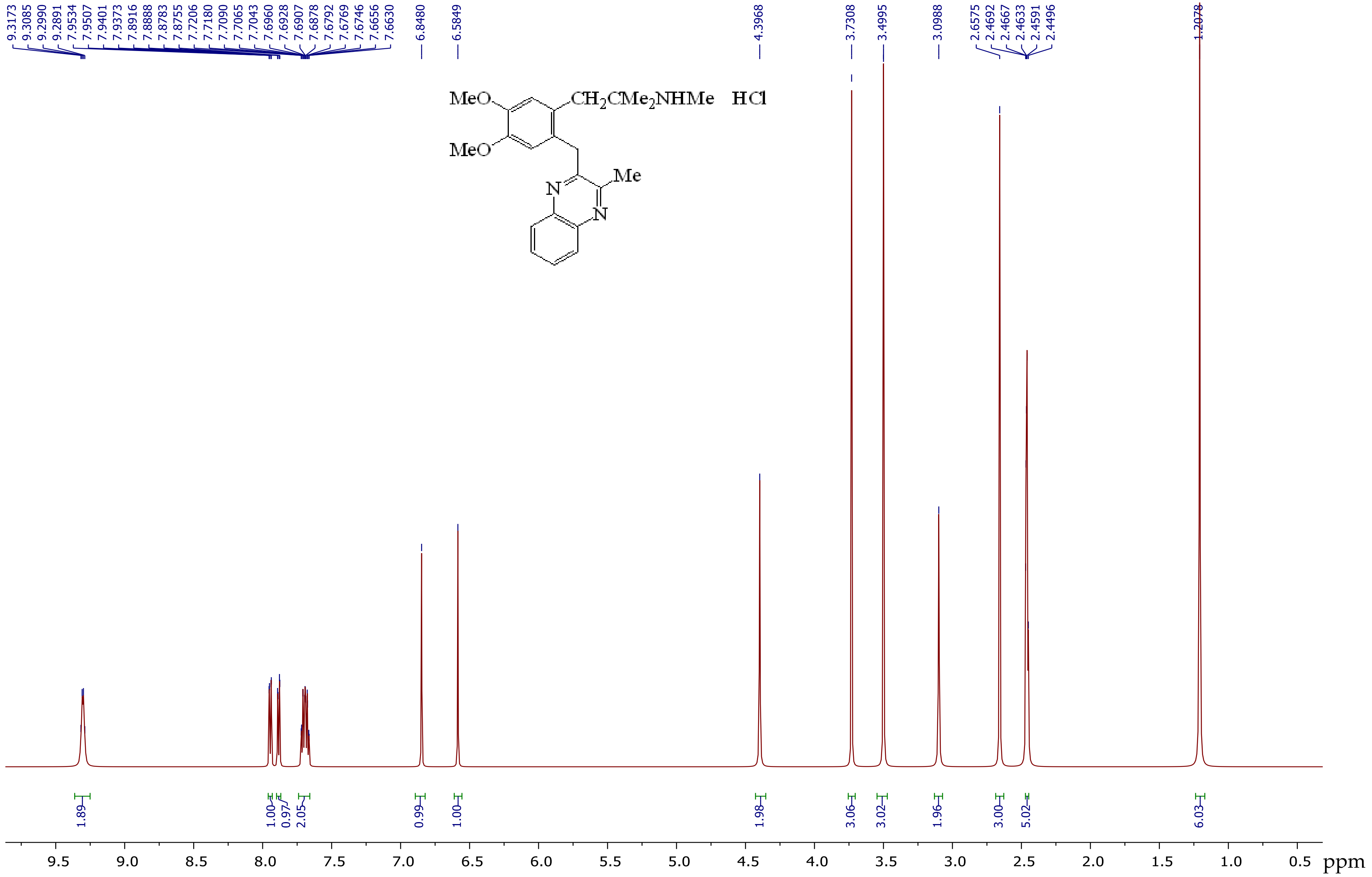

^{13}C NMR spectrum

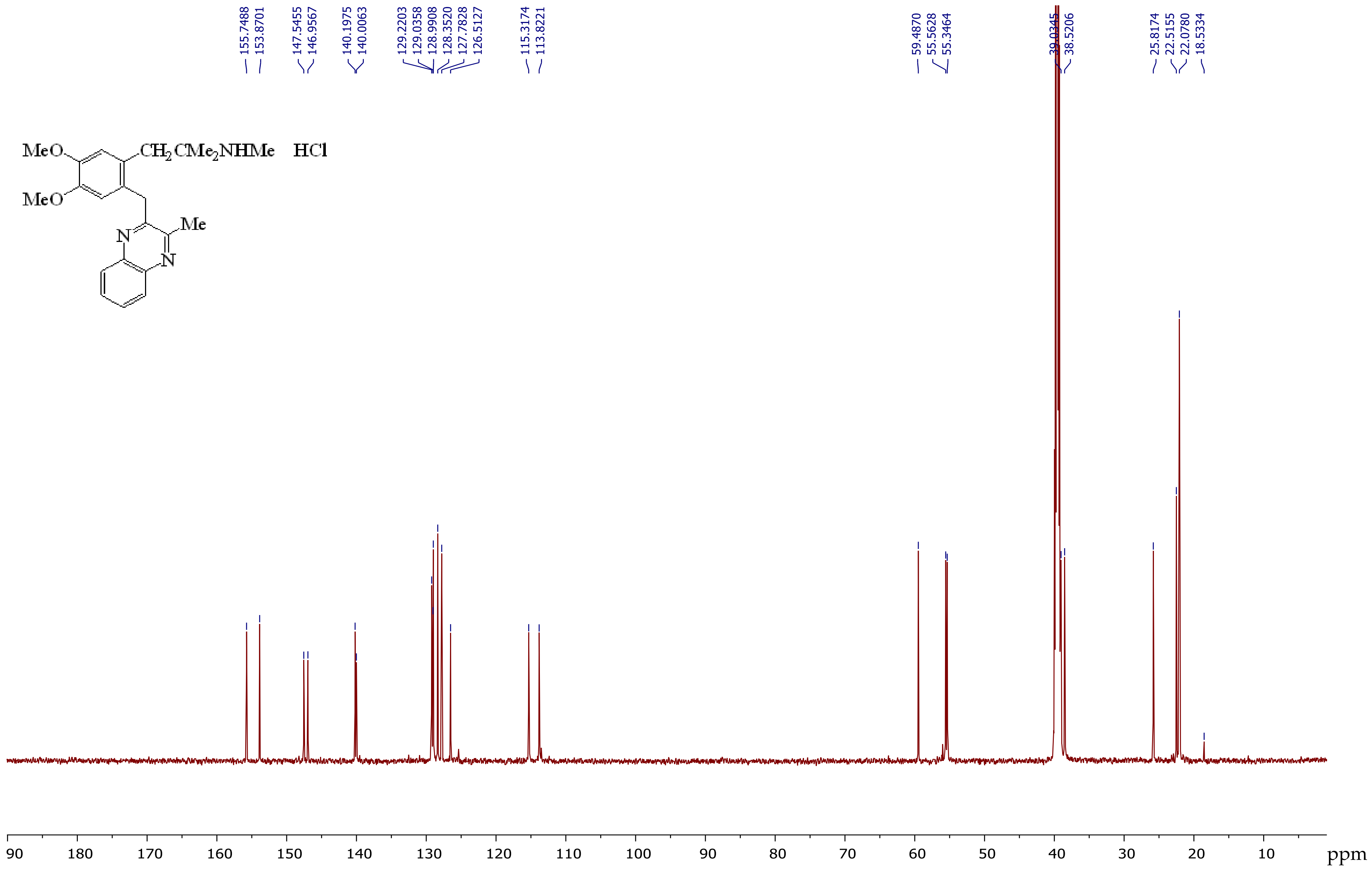

1-(2-((3-(4-bromophenyl)quinoxalin-2-yl)methyl)phenyl)-N,2-dimethylpropan-2-amine hydrochloride (**2g**):

^{1}H NMR spectrum

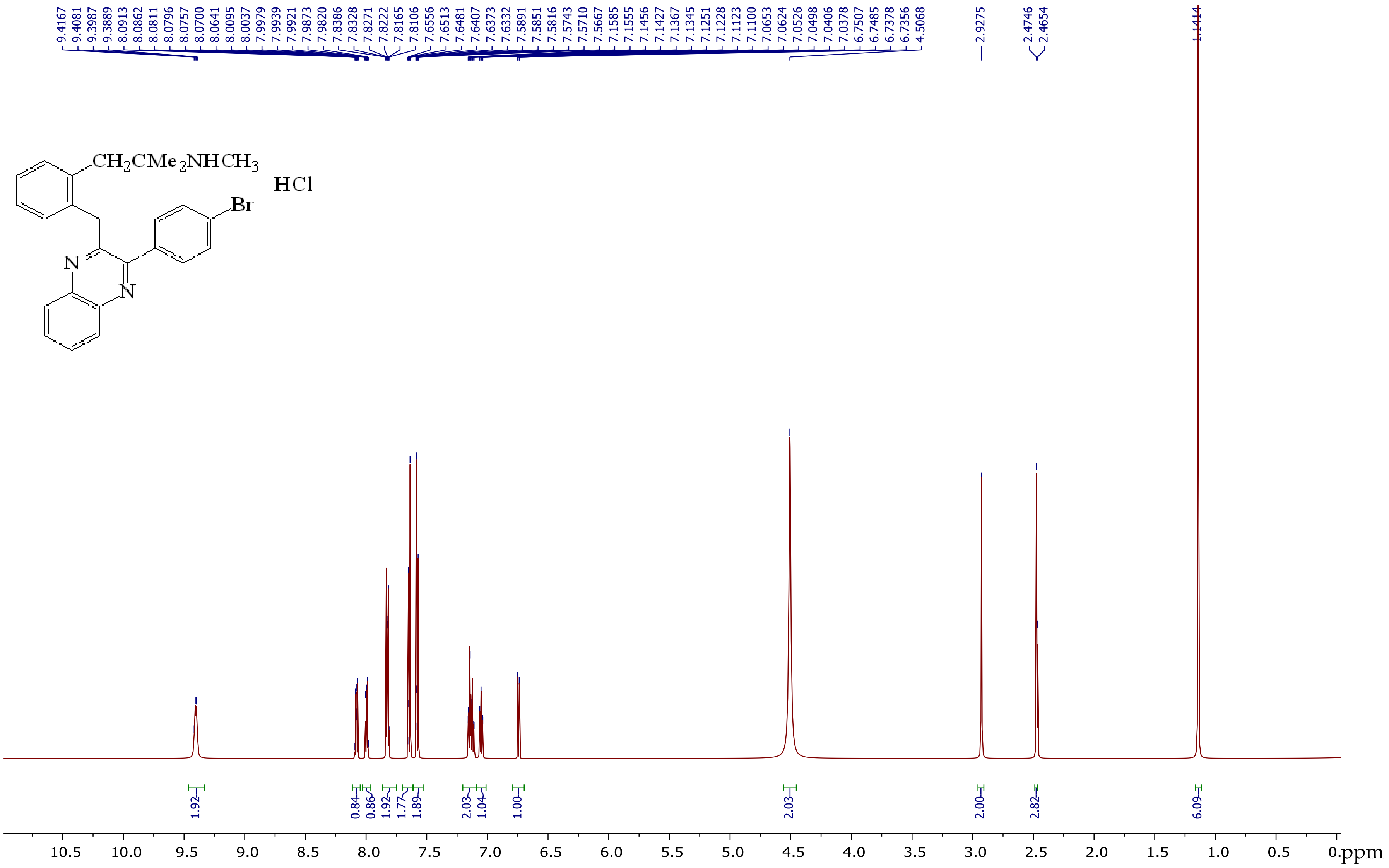

^{13}C NMR spectrum

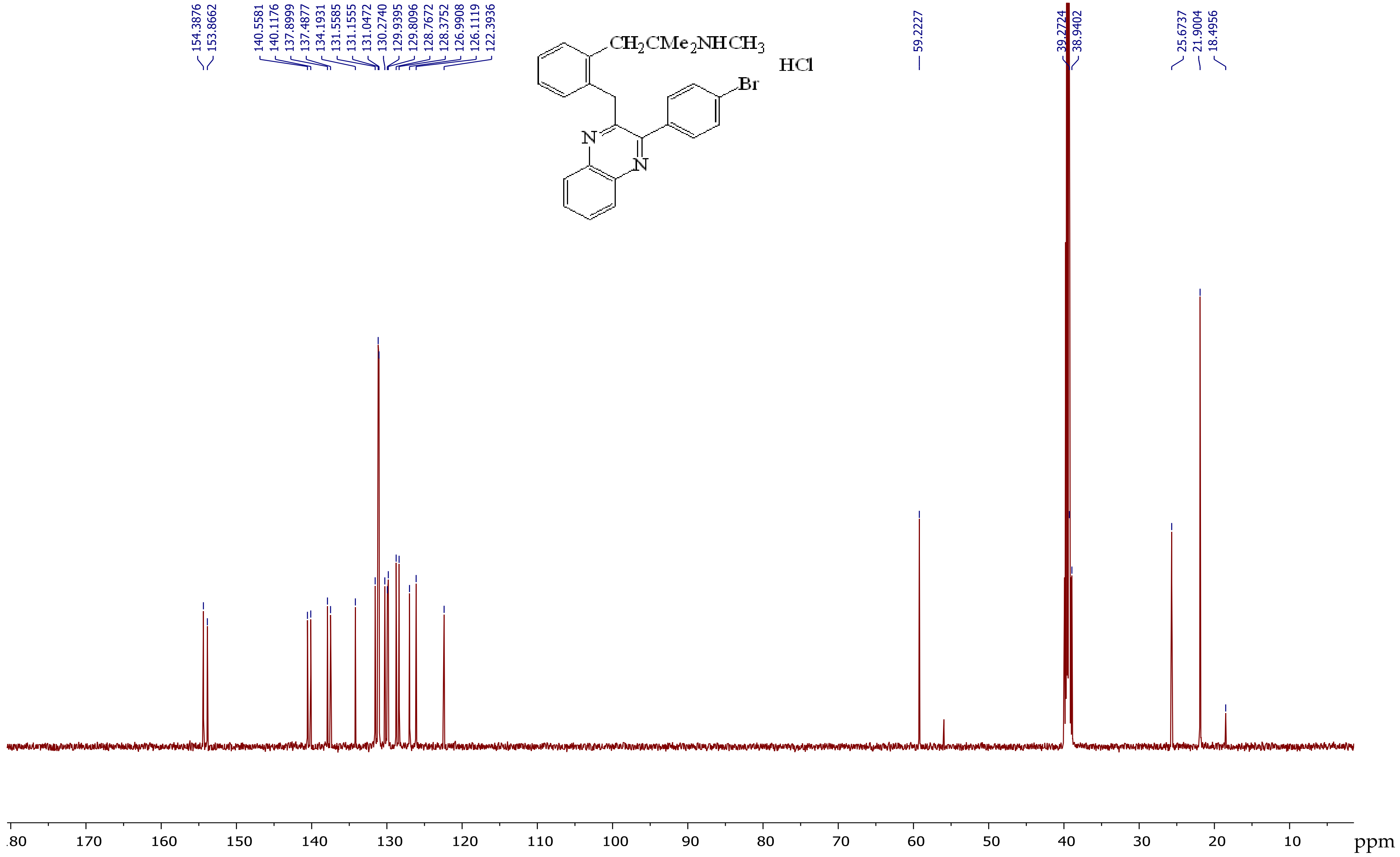

2-(2-((3-(3,4-Dichlorophenyl)quinoxalin-2-yl)methyl)-1*H*-indol-3-yl)-N-methylethan-1-amine hydrochloride (**4b**):

^{1}H NMR spectrum

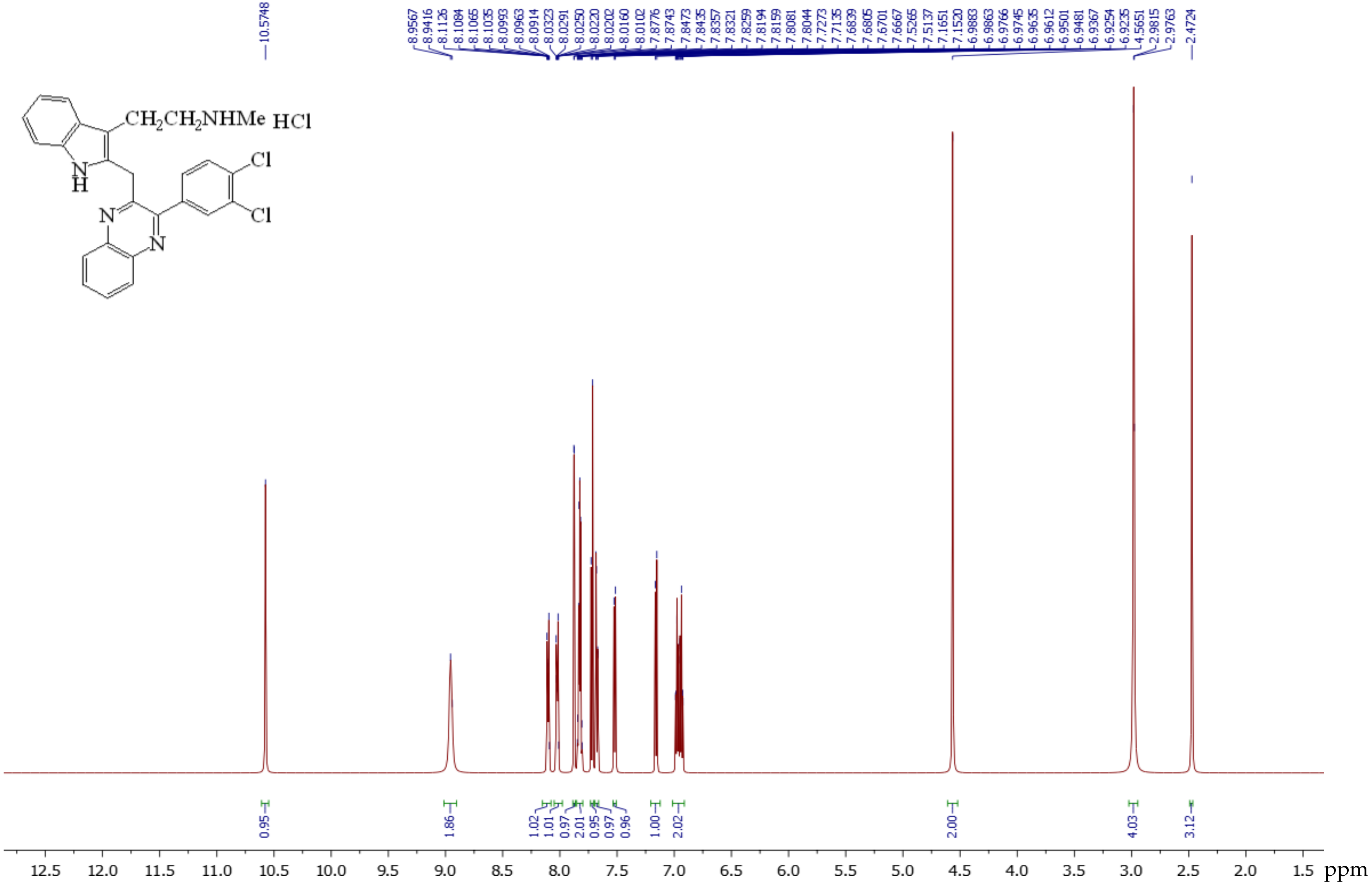

^{13}C NMR spectrum

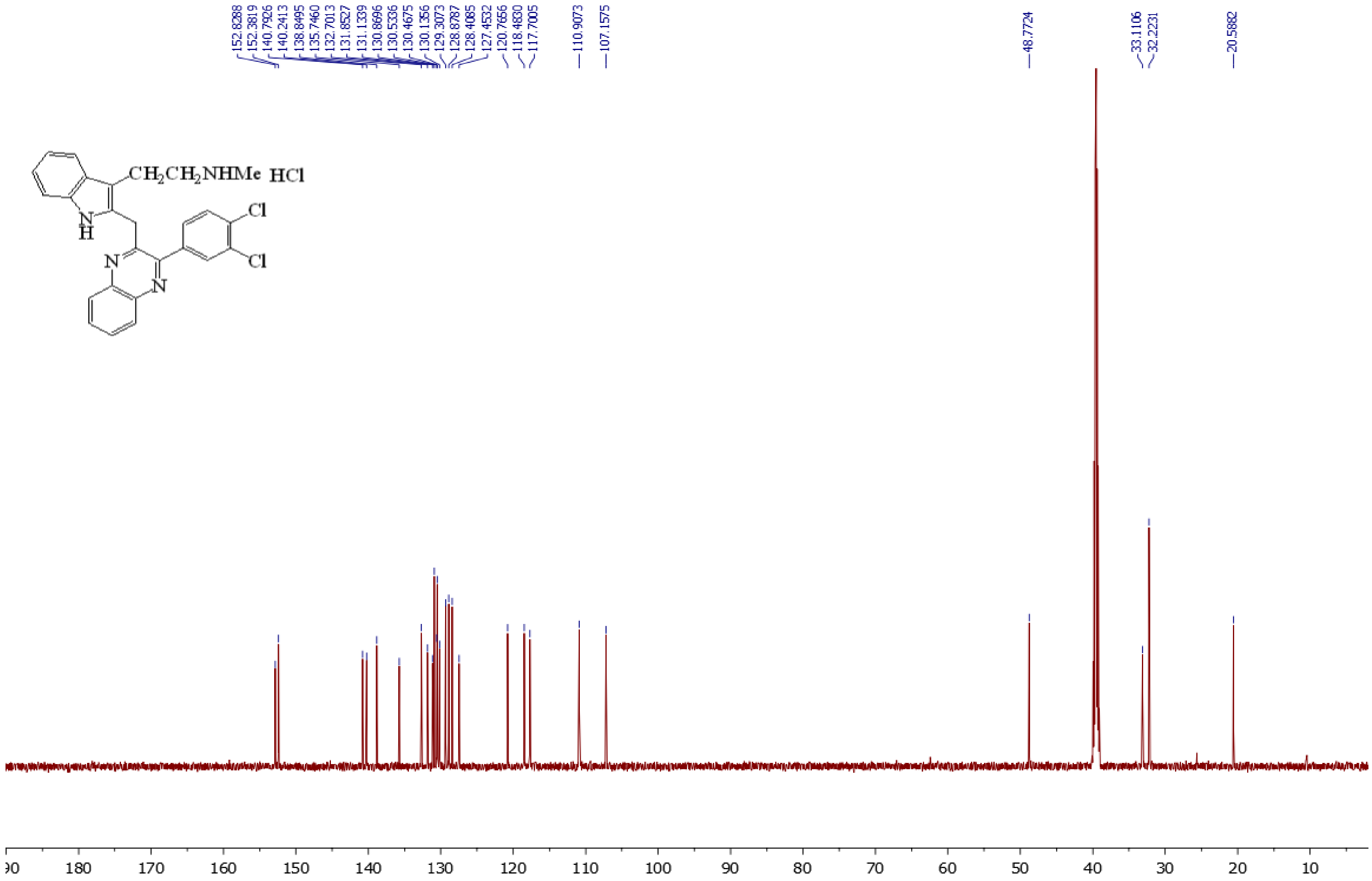

Supplement: Supplementary file 1 [file ijms-23-14401-s001.zip › ijms-1915241-supplementary.pdf]
